# Biomolecular Chirality Is Imprinted on One Layer of Hydration Water

**DOI:** 10.1021/acscentsci.2c01148

**Published:** 2022-10-06

**Authors:** Yuki Nagata, Mischa Bonn

**Affiliations:** Max Planck Institute for Polymer Research, Achermannweg 10, Mainz, 55122, Germany

One could argue that water^1^ and biomolecular chirality^2^ are crucial ingredients for comprehending the origin of life. Hence, understanding the interaction between water and chiral molecules constitutes an important and exciting endeavor. Generally speaking, biomolecular hydration plays a crucial role in determining the structure and function of biological molecules.^3^ For instance, the stabilization of protein secondary structure and the bioactivity are governed by the coupling between the protein and water. The study of biomolecular hydration is extremely challenging: biomolecule concentration is typically low, making it difficult to eliminate the large background response from water distant from the biomolecules. To shed light on the role of chirality and biomolecular hydration adds an additional level of complexity. For chiral molecules specifically, a recurring and important question is how much of the chirality is imprinted on the hydration water.

**Figure 1:**
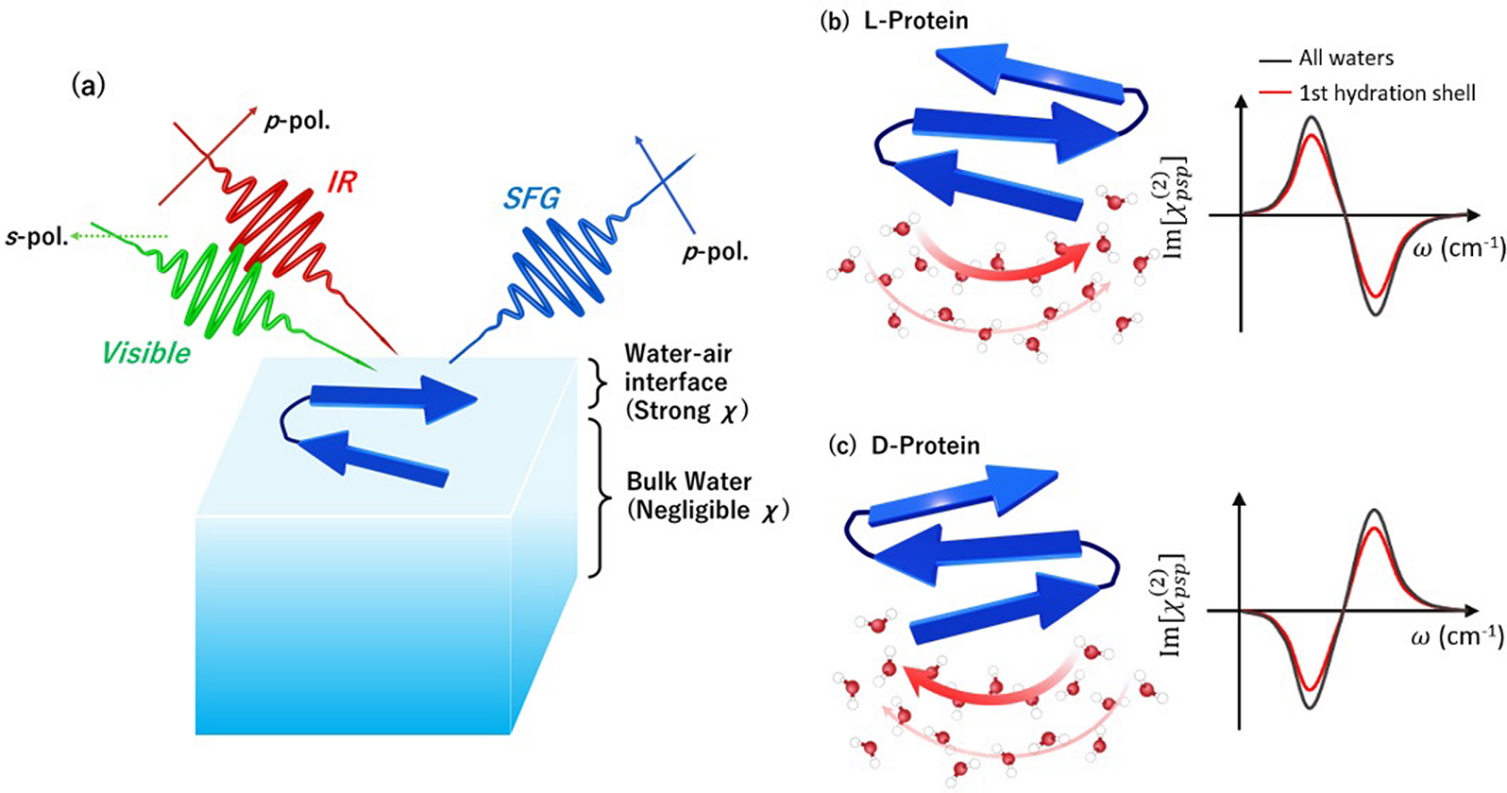
(a) Schematic of chiral SFG at the *psp* polarization combination, where *p*-, *s*-, *p*- represent the polarization of IR, visible, and SFG pulses. The blue arrows represent the orientation of protein with an antiparallel β-sheet structure located at the water–air interface. Schematics of (b) l- and (c) d-chiral protein structure and the hydrated molecules. The collective chirality is highlighted by the red arrows. The expected χ^(2)^ spectra of water at the *psp* polarization combination are drawn.

Against these odds, in this issue of *ACS Central Science*, Konstantinovsky et al. have successfully probed the protein-induced chirality of hydration water. Protein-induced chirality of water arises from the “collective”, supramolecular chirality of the hydrated water molecules, rather than from the single-molecule chirality of water. On average, the hydrated water molecules may be randomly oriented at room temperature, but the net chirality does not necessarily vanish. Combining experiments with advanced molecular modeling, the authors determined that the chirality imprint on the hydration shell is limited to precisely one layer of water. To suppress the overwhelming background signal from bulk water and to obtain a probe specifically for hydration water, the authors took a counterintuitive approach: they studied chiral proteins at the interface of water and air. This is counterintuitive because the absolute number of protein and water molecules in one monolayer of hydrated proteins at the interface is exceedingly low. Yet, by probing the proteins and water at the interface, Konstantinovsky et al. could use highly sensitive, surface-specific second-order nonlinear optical spectroscopy, namely, sum-frequency generation (SFG) spectroscopy.^4^ An SFG signal is generated by the infrared and visible pulses and enhanced when the infrared frequency is resonant with the molecular vibrations. This provides the molecular specificity to the SFG signal. Previous studies have shown that chiral SFG signals originate from the interface (Figure 1a).^5,6^ However, such SFG signals are still not sufficient to probe the water near biomolecules because  does not clearly state where the signal of water arises from. When the protein is present at the interface, the interface is very heterogeneous, and thus the SFG signal can tell us only the averaged signal at the interfacial region.

The paper by Konstantinovsky et al. establishes an elegant method to probe the first hydration shell of water near the interfacial biomolecules through the unique combination of polarized lights used in the SFG measurement. In their study, Konstantinovsky et al. use chiral SFG spectroscopy.^5^ In chiral SFG, the signal can arise solely from the interfacial molecules which possess chirality. In particular, in the heterodyne detected chiral SFG, the sign of the  feature is flipped when the chirality is flipped (Figure 1b,c).^8^ When the chirality is imprinted by the biomolecules onto the hydration water, water’s chiral SFG signal becomes nonzero. Interestingly, the SFG spectra simulation shows that such a chiral imprint is limited strictly to the first hydration shell, and the collective chirality of the water molecules is not encoded beyond the first layer. This finding guarantees that the first hydration shell can be probed with chiral SFG (Figure 1b,c). As such, the heterodyne-detected chiral SFG spectroscopy of water possesses interface specificity, molecular specificity, and first-hydration shell specificity.

The work in ref (7) highlights the strength of combining experiments with advanced SFG spectral simulation. The simulations reproduce both the achiral and the chiral response. One of the key advantages of the simulation of the vibrational spectra is the ability to disentangle the spectral contribution; for example, the different water contributions to the overall spectra can be disentangled into contributions near the backbone of the protein and near the side chain. This analysis allows us to quantify the nature of water at different molecular moieties.

A chiral SFG technique together with the findings disclosed by Konstantinovsky and co-workers^7^ ensures that the chirality near the DNA arises from the first hydration shell,^9^ allowing the mapping of the hydrated water molecules to the DNA structure. Furthermore, by applying the chiral SFG technique to monitoring the protein docking process, one can see how proteins are dehydrated by the docking process. This research direction will be particularly helpful in unveiling the mechanism of the protein foldings/misfoldings and rational drug design. Such an in situ measurement is now on the horizon.
